# The predictive value of infrared thermal imaging (IRT) for peripheral artery disease: A systematic review

**DOI:** 10.1097/MD.0000000000035639

**Published:** 2023-10-27

**Authors:** Teguh Marfen Djajakusumah, Valeska Siulinda Candrawinata, Jackie Pei Ho, Herry Herman, Kiki Lukman, Ronny Lesmana

**Affiliations:** a Division of Vascular and Endovascular Surgery, Department of Surgery, Faculty of Medicine, Padjadjaran University, Bandung, Indonesia; b Faculty of Medicine, Pelita Harapan University, Tangerang, Indonesia; c Department of Surgery, Yong Loo Lin School of Medicine, National University of Singapore, Singapore; d Department of Orthopaedic and Traumatology, Faculty of Medicine, Padjadjaran University, Bandung, Indonesia; e Division of Digestive Surgery, Department of Surgery, Faculty of Medicine, Padjadjaran University, Bandung, Indonesia; f Department of Physiology, Faculty of Medicine, Padjadjaran University, Bandung, Indonesia.

**Keywords:** infrared thermal imaging, perfusion, peripheral vascular disorders, prediction

## Abstract

**Background:**

Medical infrared thermal imaging (IRT) has been applied to research blood flow, breast cancer detection, and human body muscle performance. The benefits of IRT include the fact that it is noninvasive, quick, dependable, non-contact, capable of creating several recordings in a short period of time, and secure for both patients and medical professionals. We aimed to determine the predictive value of IRT for identifying and evaluating any interventional procedure in patients affected by peripheral artery disease (PAD) of any severity.

**Methods:**

We searched the Cochrane Library, EMBASE, and PubMed on the topic of IRT and PAD until January 20,2023. We excluded gray literature as it is lacking credibility for not undergoing a peer-reviewed process. The search strategy includes the medical topic headings for “infrared thermal imaging” and “peripheral vascular disorders.” The primary outcome of this systematic review was the variation in tissue perfusion in PAD patients. Each technique’s technical characteristics and therapeutic use within PAD must be described in each included study.

**Results:**

This systematic review included 2 case reports and 3 observational studies. By comparing the temperatures of PAD patients hands, legs, and feet, IRT might prove to be an unduly valuable tool for treating vascular illnesses, especially in light of the knowledge gained from the temperature distribution maps.

**Conclusion:**

This noninvasive method demonstrated encouraging results in the detection of various areas of foot perfusion and the screening of PAD, and it gave good findings in gauging the effects of any type of intervention.

## 1. Introduction

Intermittent claudication, rest discomfort, artery ulcers, and gangrene (together known as critical limb ischemia) are common manifestations of Peripheral artery disease (PAD). Ten percentage to 20% of the general population are affected by PAD, and its prevalence is rising as the population becomes older and diabetes is becoming more common.^[1]^ Physical examination and diagnostic tests are used to determine the diagnosis of PAD.^[2,3]^

The noninvasive measurement of muscle metabolism using near-infrared spectroscopy has also been used in investigations on PAD patients in different situations, both static and dynamic.^[4,5]^ This method offers data on tissue microvascular hemodynamics while monitoring the equilibrium between oxygen delivery and consumption. The technology was evaluated in diagnosing patients with PAD and for assessing foot perfusion during movement or response to or during treatments because it is simple to use for bedside measurements.^[6]^

A non-contact instrument known as infrared thermography, infrared imaging, or thermal imaging maps the surface temperature of a body or an item. Its uses range from industrial condition monitoring to medical imaging.^[1]^ The infrared thermal imaging within the medical field has been applied to research blood flow, human body, muscle performance, and breast cancer detection. The sensitivity changes in skin temperature related to specific disorders have been quantified using thermal imaging.^[7,8]^

The benefits of infrared thermography include the fact that it is not an invasive tool, quick, dependable, contactless, capable of creating several recordings immediately, and secure for both patients and medical professionals. Recent applications include the diagnosis of osteoarthritis and breast cancer and the assessment of pain.^[9]^ In clinical diagnostics, infrared imaging is used as a physiological test to measure the subtle physiological changes that may be brought on by a variety of conditions, such as deep venous thrombosis, liver disease, bacterial infections, contusions, fractures, burns, carcinomas, lymphomas, melanomas, prostate cancer, dermatological diseases, rheumatoid arthritis, diabetes mellitus, and associated pathology. Regional vasodilation, hyperthermia, hyperperfusion, hypermetabolism, and hypervascularization, which provide a higher-temperature heat source, are frequently linked to these disorders.^[10,11]^ Additionally, numerous studies have looked into the use of cutaneous temperature in the identifying if patients suffer diabetic foot, distinguishing various peripheral vascular disease, and assessing the presence of Raynaud phenomenon. However, all the evidences published were in the form of case reports.^[10–13]^

As of January 2023, we found there was only 1 publication in the review format discussing the utilization of infrared thermal imaging (IRT) within the medical domains. The paper was published a decade ago and it aims to determine the tissue perfusion and IRT advances in medicinal advantages for PAD patients.^[14]^ Henceforth, we aimed to determine the predictive value of IRT for identifying and evaluating any experimental technique in patients with PAD of any degree.

## 2. Methods

### 2.1. Database and literature search

This review has been registered at the international prospective register of systematic reviews under the registration number CRD42023403063. We searched the Cochrane Library, EMBASE, and PubMed for relevant papers. We excluded gray literature as it is lacking credibility for not undergoing peer-reviewed process. The search strategy includes the medical topic headings for “infrared thermal imaging” and “peripheral vascular disorders”. Detailed search queries form each electronic sources are displayed in Table 1. We described the predictive value of IRT for identifying and evaluating any experimental technique in patients with PAD of any degree from eligible studies. We were not limited to any language. We conducted the research search from January 6 until March 1, 2023. The initial search and screening were done by 2 independent authors. Any discrepancies were resolved by discussion with other authors.

**Table 1:** Detailed search terms used from Cochrane Library, EMBASE, and PubMed.

Medical database	Search queries	Studies retrieved
Cochrane Library	Thermal imaging OR infrared thermal imaging AND perfusion OR revascularization AND peripheral artery disease OR peripheral vascular disease OR arterial occlusive disease OR critical limb ischemia	149
EMBASE	Thermal imaging OR Infrared thermal imaging AND perfusion OR revascularization AND peripheral artery disease OR peripheral vascular disease OR arterial occlusive disease OR critical limb ischemia	127
PubMed	(((“thermal”[All Fields] OR “thermalization”[All Fields] OR “thermalize”[All Fields] OR “thermalized”[All Fields] OR “thermalizes”[All Fields] OR “thermalizing”[All Fields] OR “thermally”[All Fields] OR “thermals”[All Fields]) AND (“image”[All Fields] OR “image s”[All Fields] OR “imaged”[All Fields] OR “imager”[All Fields] OR “imager s”[All Fields] OR “imagers”[All Fields] OR “images”[All Fields] OR “imaging”[All Fields] OR “imaging s”[All Fields] OR “imagings”[All Fields])) OR (“infrared”[All Fields] AND (“thermal”[All Fields] OR “thermalization”[All Fields] OR “thermalize”[All Fields] OR “thermalized”[All Fields] OR “thermalizes”[All Fields] OR “thermalizing”[All Fields] OR “thermally”[All Fields] OR “thermals”[All Fields]) AND (“image”[All Fields] OR “image s”[All Fields] OR “imaged”[All Fields] OR “imager”[All Fields] OR “imager s”[All Fields] OR “imagers”[All Fields] OR “images”[All Fields] OR “imaging”[All Fields] OR “imaging s”[All Fields] OR “imagings”[All Fields]))) AND (“perfusable”[All Fields] OR “perfusate”[All Fields] OR “perfusates”[All Fields] OR “perfuse”[All Fields] OR “perfused”[All Fields] OR “perfuses”[All Fields] OR “perfusing”[All Fields] OR “perfusion”[MeSH Terms] OR “perfusion”[All Fields] OR “perfusions”[All Fields] OR (“revascularisation”[All Fields] OR “revascularisations”[All Fields] OR “revascularise”[All Fields] OR “revascularised”[All Fields] OR “revascularising”[All Fields] OR “revascularization”[All Fields] OR “revascularizations”[All Fields] OR “revascularize”[All Fields] OR “revascularized”[All Fields] OR “revascularizes”[All Fields] OR “revascularizing”[All Fields])) AND (“peripheral arterial disease”[MeSH Terms] OR (“peripheral”[All Fields] AND “arterial”[All Fields] AND “disease”[All Fields]) OR “peripheral arterial disease”[All Fields] OR (“peripheral”[All Fields] AND “artery”[All Fields] AND “disease”[All Fields]) OR “peripheral artery disease”[All Fields] OR (“peripheral vascular diseases”[MeSH Terms] OR (“peripheral”[All Fields] AND “vascular”[All Fields] AND “diseases”[All Fields]) OR “peripheral vascular diseases”[All Fields] OR (“peripheral”[All Fields] AND “vascular”[All Fields] AND “disease”[All Fields]) OR “peripheral vascular disease”[All Fields]) OR (“arterial occlusive diseases”[MeSH Terms] OR (“arterial”[All Fields] AND “occlusive”[All Fields] AND “diseases”[All Fields]) OR “arterial occlusive diseases”[All Fields] OR (“arterial”[All Fields] AND “occlusive”[All Fields] AND “disease”[All Fields]) OR “arterial occlusive disease”[All Fields]) OR (“chronic limb threatening ischemia”[MeSH Terms] OR (“chronic”[All Fields] AND “limb threatening”[All Fields] AND “ischemia”[All Fields]) OR “chronic limb threatening ischemia”[All Fields] OR (“critical”[All Fields] AND “limb”[All Fields] AND “ischemia”[All Fields]) OR “critical limb ischemia”[All Fields]))	31

MeSH = medical topic headings.

### 2.2. Study selection

Articles were included in this systematic review if they fulfilled the criteria as follows: Population: adult patients with any degree of PAD; Intervention: evaluation using IRT; Comparison: None; Outcome: the variation in tissue perfusion; Type of studies: observational studies and case reports. We excluded review articles, correspondences and editorials. Tissue perfusion status is defined by any diagnostic test performed to assess peripheral perfusion, such as ankle-brachial index, laser doppler blood flow, and others. The tissue perfusion status must be obtained before the patients receiving any treatment. Each technique technical characteristics and therapeutic use within PAD must be described from each included study. Studies that did not provide the were excluded.

### 2.3. Data extraction and quality assessment

Each reviewer separately extracted the data onto an electronic spreadsheet we had appropriately constructed. The information gathered covered publication information, such as principal investigator, date of publication, country of which the study was conducted, the design of the study, cohort characteristics, the procedural details of where and how IRT was measured, the cutoff value, precision details of IRT, timing of measurements, and other detailed outcomes. The cohort characteristics extracted from each studies include patients age, sex, total cohort, and the degree of PAD.

We evaluated the research applicability and risk of bias using the Joanna Briggs Institute checklist for case reports and Newcastle Ottawa scale for observational studies. When quality domains are present, the authors grants points according to the Newcastle Ottawa scale criteria, allowing the computation of overall “quality scores”.^[15,16]^

This study is reported in line with the Preferred Reporting Items for Systematic Reviews and Meta-Analyses guidelines.^[17]^

## 3. Results

**Figure 1.**
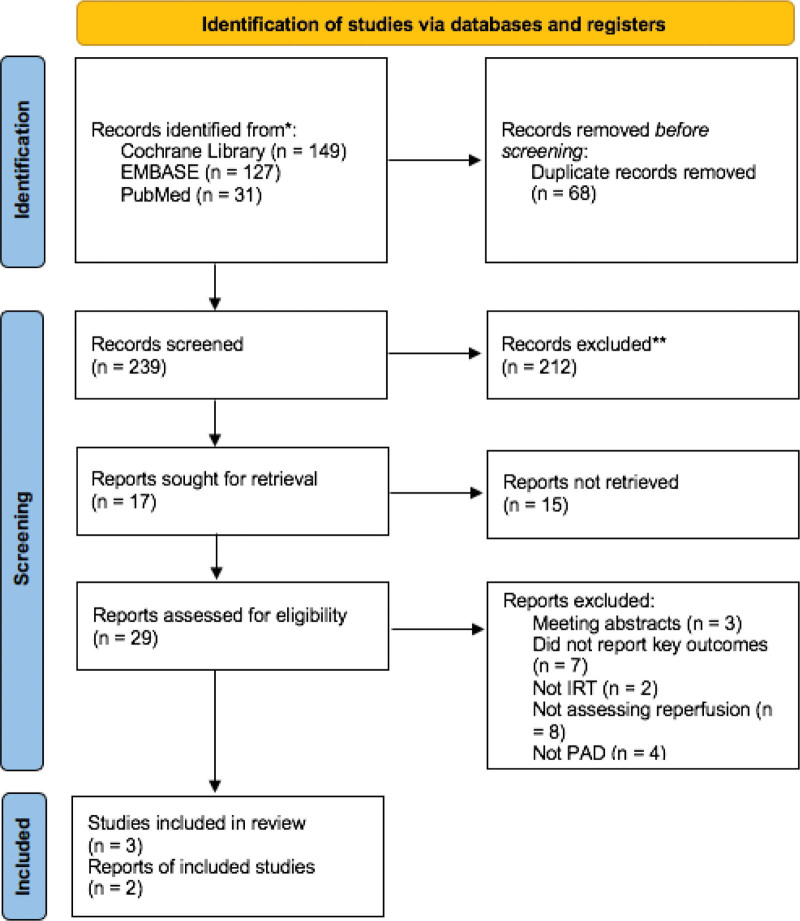
PRISMA flow diagram. PRISMA = preferred reporting items for systematic reviews and meta-analyses.

We identified a total of 307 publications as a result of the database queries, 68 of which were duplicates. We excluded 212 articles after being subjected to title and abstract screening in accordance with the inclusion criteria. After screening the entire texts of the remaining 29 papers, 24 articles were eliminated. Finally, it was determined that 5 articles qualified for inclusion.^[18–22]^ Figure 1 displays the diagram of the study’s flow.

**Figure 2.**
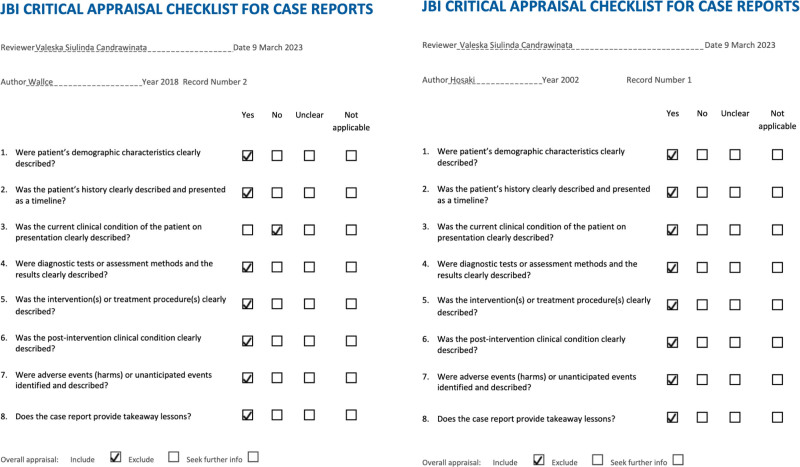
Critical appraisal for included case reports.

This systematic review included 2 case reports and 3 observational studies. For the primary outcome, we found that IRT is a noninvasive device and has the potential in detecting peripheral perfusion and the screening of PAD, and in gauging the effects of any type of intervention. By comparing the temperatures of PAD patients’ hands, legs, and feet, Wang et al analyzed blood circulation. We noted significant temperature changes at the foot, particularly the toes. IRT might prove to be an unduly valuable tool for treating vascular illnesses, especially considering the knowledge gained from the temperature distribution maps. The early stages of PAD and diabetic mellitus (DM) patients were the focus of Hosaki et al^[19]^ investigation, and they discovered that IRT could be helpful as a noninvasive tool to quickly identify patients with poor peripheral circulation for the assessment of further treatment. Gatt et al^[20]^ employed IRT to examine how patients with DM, either with or without PAD, had different foot temperatures. The patients with concurrent DM and PAD had a greater temperature than those with DM alone, according to the authors observations of a significant difference in temperature at each toe. The summary of included studies is in Table 2. In terms of quality assessment, we found that the 3 observational studies and 2 case reports were of good quality. Detailed quality assessment is shown in Table 3 and Figure 2.

**Table 2:** Summary of data from each eligible study.

Study ID	Study design	Stage of PAD	Total cohort (n)	Age (years, median [range])	IRT details	Range of temperature	Location of measurement	Perfusion parameters
Wallace 2018	Case report	I–IV	23	66 (30–76)	Dividing the maximum foot temperature by the maximum temperature recorded in both hands	-	Hands	ABI
Hosaki 2002	Case report	-	27	67.4 (51–82)	Using the image processing tools, BSA at temperatures above the selected baseline of 27°C was estimated.	-	Feet	LDBF
Gatt 2018	Observational study	I–II	182	-	Both visual and thermal photos were captured throughout the patient’s 15-minute acclimatization period while they were laying supine in a 23°C-controlled clinic. The thermal and digital cameras were both positioned 1.5 m apart from the patient’s foot.	-	Feet	ABI, SD, 10 g monofilament
Wang 2004	Observational study	-	7	-	A 256 × 256 InSb FPA detector is built within the camera. Using a 25 mm and 50 mm lens, the temperature resolution is 0.015K (12-bit digital output), and the complete frame speed is 144Hz.	-	Ankle, Feet	No parameters
Staffa 2016	Observational study	I–III	41	60.2 ± 18	One meter away from the body area, thermal pictures were taken in a constrained, temperature-controlled setting. 15 minutes long.	+0.4 °C (treated); −0.5 °C (non-treated)	Feet	ABI

ABI = ankle-brachial index, BSA = body surface area, IRT = infrared thermal imaging, LDBF = laser doppler blood flow, PAD = peripheral artery disease.

**Table 3:** NOS assessment for risk of bias.

Study ID	Gatt 2018	Wang 2004	Staffa 2016
Selection			
Representativeness of the exposed cohort	*	*	*
Selection of the nonexposed cohort		*	*
Ascertainment of exposure	*		*
Demonstration that outcome of interest was not present at start of study	*	*	*
Comparability			
Comparability of cohorts on the basis of the design or analysis controlled for confounders	*	*	*
Outcome			
Assessment of outcome	*	*	*
Was follow-up long enough for outcomes to occur			
Adequacy of follow-up of cohorts			

NOS = Newcastle Ottawa scale.

Wang^[21]^ and (2004) Wallace et al^[18]^ (2018) is the first study utilizing the smart phone-based thermography and has identified good correlation between traditional and thermal ankle-brachial indices. Thermographs of each extremity were obtained, and maximum surface temperature recorded. Thermal ankle-brachial index was calculated by dividing the lower extremity temperature by the upper extremity. Whereas Hosaki (2002), Gatt (2018), and Staffa (2016) assessed the difference of foot temperature within patients suffering from type 2 diabetes mellitus and found that neuroischemic feet with or without ulceration had toe temperatures that were considerably higher.^[19,20,22]^ The temperatures of the ulcerated and nonulcerated toes did not differ significantly, suggesting that all of the toes on the same foot may be at risk of developing problems that may be seen by infrared thermography. However, all included studies population were very small and also no prospective follow-up data were available.

## 4. Discussions

Our systematic review found that IRT might prove to be an unduly valuable tool for treating vascular illnesses, especially in light of the knowledge gained from the temperature distribution maps. This noninvasive method demonstrated encouraging results in the detection of various areas of foot perfusion and the screening of PAD, and it gave good findings in gauging the effects of any type of intervention.

We learned that there are many different types of IRT devices available, each with distinctive characteristics and a range of resolutions, spectrum, and specified temperature, prices, and accuracy levels. Given that the improvement after revascularization was observed between 1- to 3- degree Celsius and that 2-degree Celsius comprises a significant fraction of the body’s temperature, most devices specifically mention a considerable margin for error. Additionally, the measurement was obtained from numerous points of reference, including the shin, ankle, foot dorsum, toes, and foot plant. Another issue that needs to be addressed is the varied measuring circumstances, in particular the room temperature and the length of time that bare feet are exposed to the room temperature. It is apparent that the temperature in the operating theater is lower than at the clinic, and that a calculation after 1 or 5 minutes of bed rest could affect different results. Finally, it is crucial to know that IRT still cannot be used for diagnosing PAD because its validated cutoff value has not yet been determined. This is due to the differences in each individual’s average skin temperature.

On the other hand, IRT offers several benefits that make it an attractive option for PAD patients. These advantages include the inexpensive cost of IRT in comparison to other diagnostic devices, the ease and quickness of data collection, and the precision and consistency of the measurements collected. It is important to take advantage of all these positive attributes with different disciplines, such as the manufacturer and physicians, in order to reduce the margin of error of the devices, planning appropriate experiments, assessing their diagnostic capability, and determine the reference points for data collection, and measurement conditions.

The following are some of the drawbacks of IRT: despite being the oldest medical imaging modality, it is not widely utilized by clinical professionals. IRT provides only temperature information, which is an indirect form of core physiological information. It also has limited accuracy of the overall temperature reading of the equipment, necessitating a controlled environment. On the other hand, IRT is a quick, simple, and relatively inexpensive imaging modality; non-contact, noninvasive, and nonionizing technique that enables imaging of large areas of the skin surface, enables real-time physiology monitoring, enables an indirect assessment of blood flow, and enables large-scale screening.

Future research on the IRT should carefully describe the subject information, previous instructions, environmental setup, equipment, acclimation, camera preparation, extrinsic factors, camera position, emissivity value, body position, and image evaluation. It is encouraged to use big samples (more than 50 subjects), a control group, and advanced statistics.

## 5. Conclusion

This systematic study, which only examines the application of IRT for patients who suffer PAD, concludes that IRT is a noninvasive method demonstrated encouraging results, especially in the recognition of early progression of PAD at various areas of the extremities. Otherwise, there is still insufficient data to support the use of IRT as the sole technique for assessing patients with PAD. To advocate the use of IRT in current vascular guidelines, properly planned, prospective and interventional trials with a lengthy follow-up duration are required.

## Author contributions

**Conceptualization:** Teguh Marfen Djajakusumah, Valeska Siulinda Candrawinata, Jackie Pei Ho, Herry Herman, Kiki Lukman, Ronny Lesmana.

**Data curation:** Teguh Marfen Djajakusumah, Valeska Siulinda Candrawinata, Jackie Pei Ho, Herry Herman, Kiki Lukman, Ronny Lesmana.

**Formal analysis:** Teguh Marfen Djajakusumah, Valeska Siulinda Candrawinata, Jackie Pei Ho, Herry Herman, Kiki Lukman, Ronny Lesmana.

**Investigation:** Teguh Marfen Djajakusumah, Valeska Siulinda Candrawinata, Jackie Pei Ho, Herry Herman, Kiki Lukman, Ronny Lesmana.

**Methodology:** Teguh Marfen Djajakusumah, Valeska Siulinda Candrawinata, Jackie Pei Ho, Herry Herman, Kiki Lukman, Ronny Lesmana.

**Project administration:** Teguh Marfen Djajakusumah, Valeska Siulinda Candrawinata, Jackie Pei Ho, Herry Herman, Kiki Lukman, Ronny Lesmana.

**Resources:** Teguh Marfen Djajakusumah, Valeska Siulinda Candrawinata, Jackie Pei Ho, Herry Herman, Kiki Lukman, Ronny Lesmana.

**Software:** Teguh Marfen Djajakusumah, Valeska Siulinda Candrawinata, Jackie Pei Ho, Herry Herman, Kiki Lukman, Ronny Lesmana.

**Supervision:** Teguh Marfen Djajakusumah, Valeska Siulinda Candrawinata, Jackie Pei Ho, Herry Herman, Kiki Lukman, Ronny Lesmana.

**Validation:** Teguh Marfen Djajakusumah, Valeska Siulinda Candrawinata, Jackie Pei Ho, Herry Herman, Kiki Lukman, Ronny Lesmana.

**Visualization:** Teguh Marfen Djajakusumah, Valeska Siulinda Candrawinata, Jackie Pei Ho, Herry Herman, Kiki Lukman, Ronny Lesmana.

**Writing – original draft:** Teguh Marfen Djajakusumah, Valeska Siulinda Candrawinata, Jackie Pei Ho, Herry Herman, Kiki Lukman, Ronny Lesmana.

**Writing – review & editing:** Teguh Marfen Djajakusumah, Valeska Siulinda Candrawinata, Jackie Pei Ho, Herry Herman, Kiki Lukman, Ronny Lesmana.
